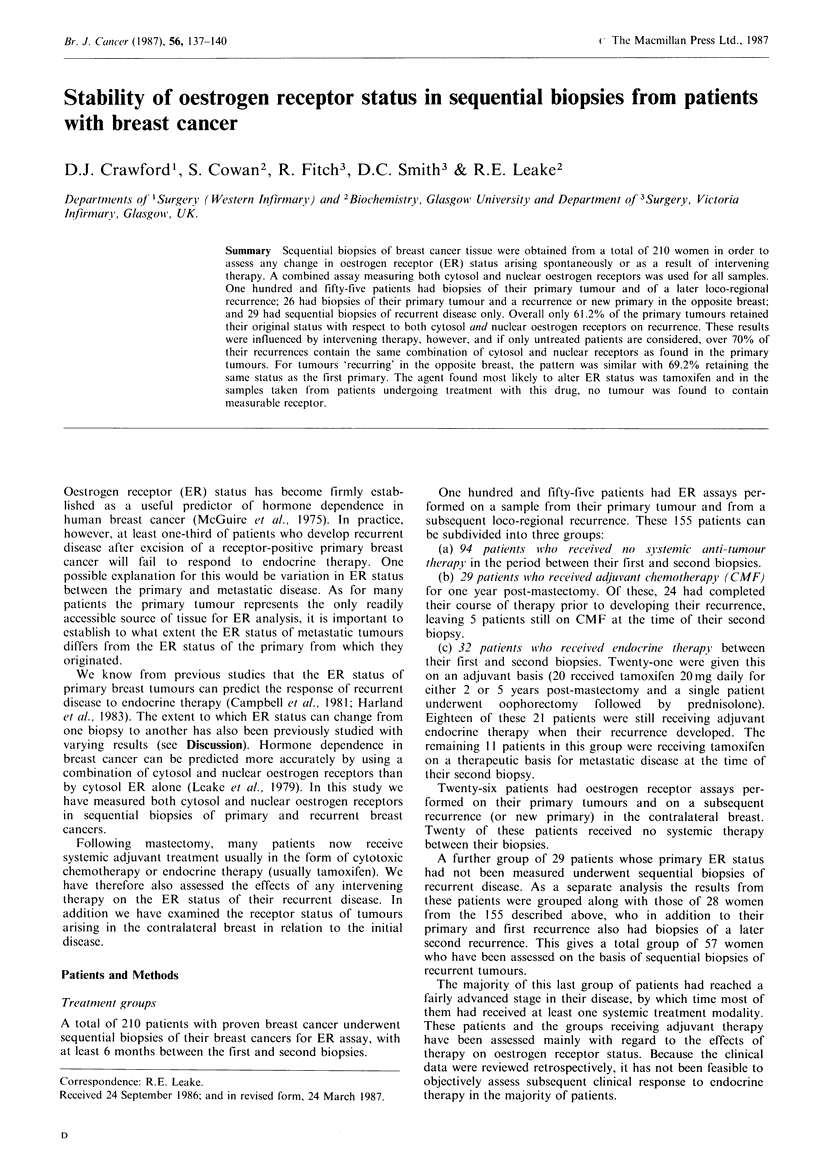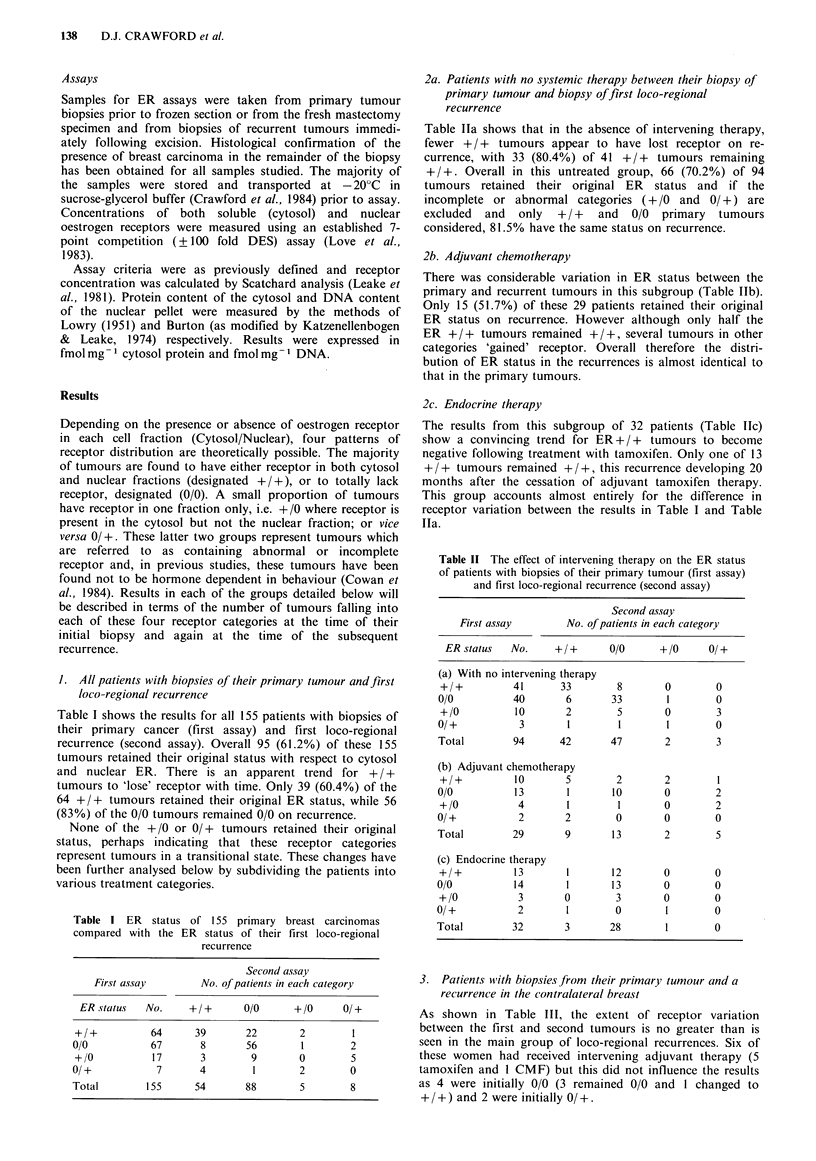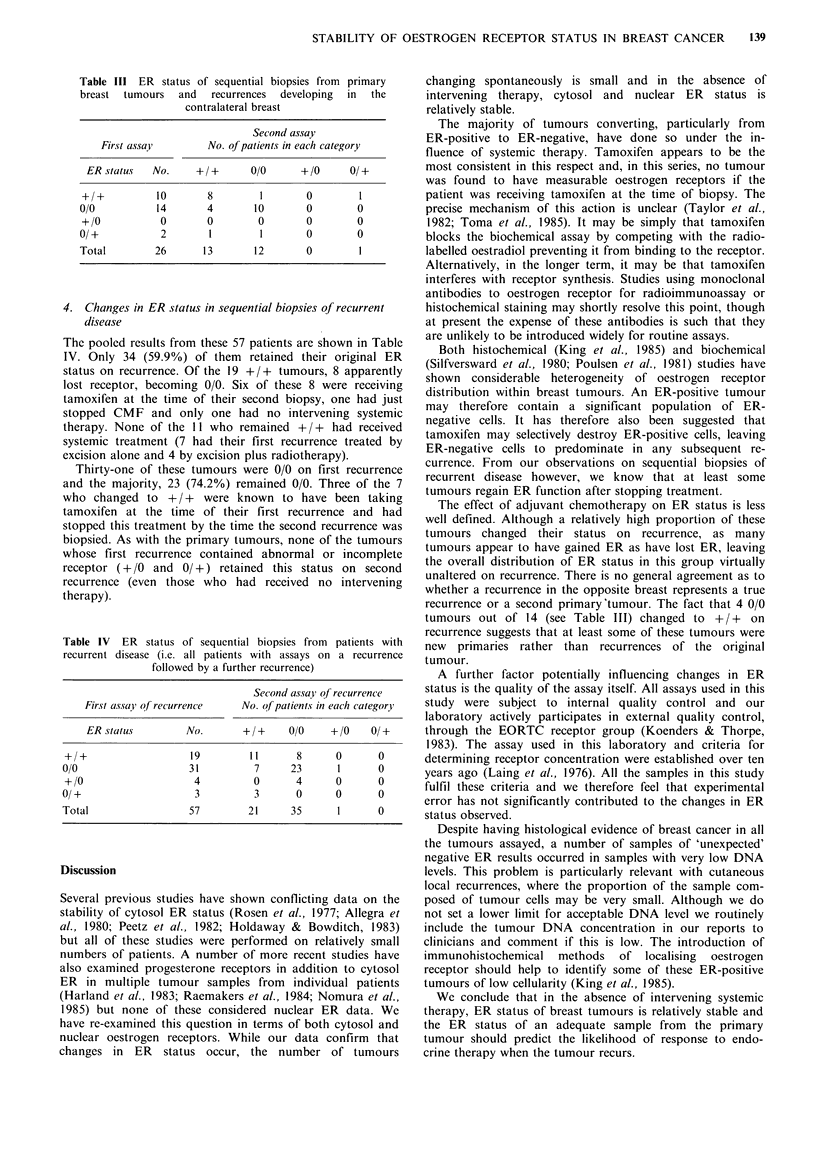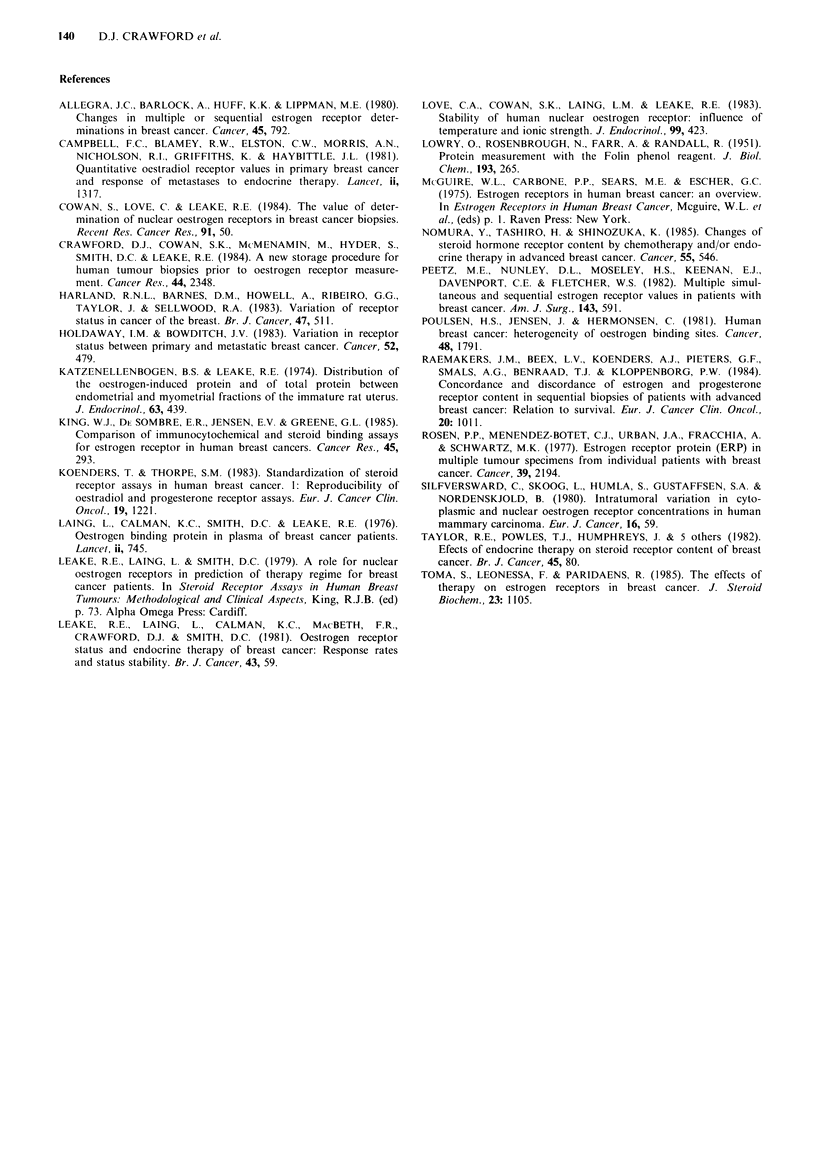# Stability of oestrogen receptor status in sequential biopsies from patients with breast cancer.

**DOI:** 10.1038/bjc.1987.171

**Published:** 1987-08

**Authors:** D. J. Crawford, S. Cowan, R. Fitch, D. C. Smith, R. E. Leake

**Affiliations:** Department of Surgery (Western Infirmary), Glasgow University, UK.

## Abstract

Sequential biopsies of breast cancer tissue were obtained from a total of 210 women in order to assess any change in oestrogen receptor (ER) status arising spontaneously or as a result of intervening therapy. A combined assay measuring both cytosol and nuclear oestrogen receptors was used for all samples. One hundred and fifty-five patients had biopsies of their primary tumour and of a later loco-regional recurrence; 26 had biopsies of their primary tumour and a recurrence or new primary in the opposite breast; and 29 had sequential biopsies of recurrent disease only. Overall only 61.2% of the primary tumours retained their original status with respect to both cytosol and nuclear oestrogen receptors on recurrence. These results were influenced by intervening therapy, however, and if only untreated patients are considered, over 70% of their recurrences contain the same combination of cytosol and nuclear receptors as found in the primary tumours. For tumours 'recurring' in the opposite breast, the pattern was similar with 69.2% retaining the same status as the first primary. The agent found most likely to alter I:R status was tamoxifen and in the samples taken from patients undergoing treatment with this drug, no tumour was found to contain measurable receptor.


*Br. J. Cancer* (1987), **56**, 137–140

© The Macmillan Press Ltd., 1987

### Stability of oestrogen receptor status in sequential biopsies from patients with breast cancer

D.J. Crawford[1], S. Cowan[2], R. Fitch[3], D.C. Smith[3] & R.E. Leake[2]

*Departments of [1]Surgery (Western Infirmary) and [2]Biochemistry, Glasgow University and Department of [3]Surgery, Victoria Infirmary, Glasgow, UK.*

**Summary** Sequential biopsies of breast cancer tissue were obtained from a total of 210 women in order to assess any change in oestrogen receptor (ER) status arising spontaneously or as a result of intervening therapy. A combined assay measuring both cytosol and nuclear oestrogen receptors was used for all samples. One hundred and fifty-five patients had biopsies of their primary tumour and of a later loco-regional recurrence; 26 had biopsies of their primary tumour and a recurrence or new primary in the opposite breast; and 29 had sequential biopsies of recurrent disease only. Overall only 61.2% of the primary tumours retained their original status with respect to both cytosol *and* nuclear oestrogen receptors on recurrence. These results were influenced by intervening therapy, however, and if only untreated patients are considered, over 70% of their recurrences contain the same combination of cytosol and nuclear receptors as found in the primary tumours. For tumours 'recurring' in the opposite breast, the pattern was similar with 69.2% retaining the same status as the first primary. The agent found most likely to alter ER status was tamoxifen and in the samples taken from patients undergoing treatment with this drug, no tumour was found to contain measurable receptor.

Oestrogen receptor (ER) status has become firmly established as a useful predictor of hormone dependence in human breast cancer (McGuire *et al.*, 1975). In practice, however, at least one-third of patients who develop recurrent disease after excision of a receptor-positive primary breast cancer will fail to respond to endocrine therapy. One possible explanation for this would be variation in ER status between the primary and metastatic disease. As for many patients the primary tumour represents the only readily accessible source of tissue for ER analysis, it is important to establish to what extent the ER status of metastatic tumours differs from the ER status of the primary from which they originated.

We know from previous studies that the ER status of primary breast tumours can predict the response of recurrent disease to endocrine therapy (Campbell *et al.*, 1981; Harland *et al.*, 1983). The extent to which ER status can change from one biopsy to another has also been previously studied with varying results (see **Discussion**). Hormone dependence in breast cancer can be predicted more accurately by using a combination of cytosol and nuclear oestrogen receptors than by cytosol ER alone (Leake *et al.*, 1979). In this study we have measured both cytosol and nuclear oestrogen receptors in sequential biopsies of primary and recurrent breast cancers.

Following mastectomy, many patients now receive systemic adjuvant treatment usually in the form of cytotoxic chemotherapy or endocrine therapy (usually tamoxifen). We have therefore also assessed the effects of any intervening therapy on the ER status of their recurrent disease. In addition we have examined the receptor status of tumours arising in the contralateral breast in relation to the initial disease.

### Patients and Methods

### *Treatment groups*

A total of 210 patients with proven breast cancer underwent sequential biopsies of their breast cancers for ER assay, with at least 6 months between the first and second biopsies.

Correspondence: R.E. Leake.
Received 24 September 1986; and in revised form, 24 March 1987.

One hundred and fifty-five patients had ER assays performed on a sample from their primary tumour and from a subsequent loco-regional recurrence. These 155 patients can be subdivided into three groups:

(a) *94 patients who received no systemic anti-tumour therapy* in the period between their first and second biopsies.

(b) *29 patients who received adjuvant chemotherapy (CMF)* for one year post-mastectomy. Of these, 24 had completed their course of therapy prior to developing their recurrence, leaving 5 patients still on CMF at the time of their second biopsy.

(c) *32 patients who received endocrine therapy* between their first and second biopsies. Twenty-one were given this on an adjuvant basis (20 received tamoxifen 20 mg daily for either 2 or 5 years post-mastectomy and a single patient underwent oophorectomy followed by prednisolone). Eighteen of these 21 patients were still receiving adjuvant endocrine therapy when their recurrence developed. The remaining 11 patients in this group were receiving tamoxifen on a therapeutic basis for metastatic disease at the time of their second biopsy.

Twenty-six patients had oestrogen receptor assays performed on their primary tumours and on a subsequent recurrence (or new primary) in the contralateral breast. Twenty of these patients received no systemic therapy between their biopsies.

A further group of 29 patients whose primary ER status had not been measured underwent sequential biopsies of recurrent disease. As a separate analysis the results from these patients were grouped along with those of 28 women from the 155 described above, who in addition to their primary and first recurrence also had biopsies of a later second recurrence. This gives a total group of 57 women who have been assessed on the basis of sequential biopsies of recurrent tumours.

The majority of this last group of patients had reached a fairly advanced stage in their disease, by which time most of them had received at least one systemic treatment modality. These patients and the groups receiving adjuvant therapy have been assessed mainly with regard to the effects of therapy on oestrogen receptor status. Because the clinical data were reviewed retrospectively, it has not been feasible to objectively assess subsequent clinical response to endocrine therapy in the majority of patients.

*Assays*

Samples for ER assays were taken from primary tumour biopsies prior to frozen section or from the fresh mastectomy specimen and from biopsies of recurrent tumours immediately following excision. Histological confirmation of the presence of breast carcinoma in the remainder of the biopsy has been obtained for all samples studied. The majority of the samples were stored and transported at −20°C in sucrose-glycerol buffer (Crawford *et al.*, 1984) prior to assay. Concentrations of both soluble (cytosol) and nuclear oestrogen receptors were measured using an established 7-point competition (±100 fold DES) assay (Love *et al.*, 1983).

Assay criteria were as previously defined and receptor concentration was calculated by Scatchard analysis (Leake *et al.*, 1981). Protein content of the cytosol and DNA content of the nuclear pellet were measured by the methods of Lowry (1951) and Burton (as modified by Katzenellenbogen & Leake, 1974) respectively. Results were expressed in $\text{fmol}\,\text{mg}^{-1}$ cytosol protein and $\text{fmol}\,\text{mg}^{-1}$ DNA.

### Results

Depending on the presence or absence of oestrogen receptor in each cell fraction (Cytosol/Nuclear), four patterns of receptor distribution are theoretically possible. The majority of tumours are found to have either receptor in both cytosol and nuclear fractions (designated +/+), or to totally lack receptor, designated (0/0). A small proportion of tumours have receptor in one fraction only, i.e. +/0 where receptor is present in the cytosol but not the nuclear fraction; or *vice versa* 0/+. These latter two groups represent tumours which are referred to as containing abnormal or incomplete receptor and, in previous studies, these tumours have been found not to be hormone dependent in behaviour (Cowan *et al.*, 1984). Results in each of the groups detailed below will be described in terms of the number of tumours falling into each of these four receptor categories at the time of their initial biopsy and again at the time of the subsequent recurrence.

*1. All patients with biopsies of their primary tumour and first loco-regional recurrence*

Table I shows the results for all 155 patients with biopsies of their primary cancer (first assay) and first loco-regional recurrence (second assay). Overall 95 (61.2%) of these 155 tumours retained their original status with respect to cytosol and nuclear ER. There is an apparent trend for +/+ tumours to 'lose' receptor with time. Only 39 (60.4%) of the 64 +/+ tumours retained their original ER status, while 56 (83%) of the 0/0 tumours remained 0/0 on recurrence.

None of the +/0 or 0/+ tumours retained their original status, perhaps indicating that these receptor categories represent tumours in a transitional state. These changes have been further analysed below by subdividing the patients into various treatment categories.

**Table I** ER status of 155 primary breast carcinomas compared with the ER status of their first loco-regional recurrence

| *First assay* | | *Second assay* *No. of patients in each category* | | | |
|---|---|---|---|---|---|
| *ER status* | *No.* | +/+ | 0/0 | +/0 | 0/+ |
| +/+ | 64 | 39 | 22 | 2 | 1 |
| 0/0 | 67 | 8 | 56 | 1 | 2 |
| +/0 | 17 | 3 | 9 | 0 | 5 |
| 0/+ | 7 | 4 | 1 | 2 | 0 |
| Total | 155 | 54 | 88 | 5 | 8 |

*2a. Patients with no systemic therapy between their biopsy of primary tumour and biopsy of first loco-regional recurrence*

Table IIa shows that in the absence of intervening therapy, fewer +/+ tumours appear to have lost receptor on recurrence, with 33 (80.4%) of 41 +/+ tumours remaining +/+. Overall in this untreated group, 66 (70.2%) of 94 tumours retained their original ER status and if the incomplete or abnormal categories (+/0 and 0/+) are excluded and only +/+ and 0/0 primary tumours considered, 81.5% have the same status on recurrence.

*2b. Adjuvant chemotherapy*

There was considerable variation in ER status between the primary and recurrent tumours in this subgroup (Table IIb). Only 15 (51.7%) of these 29 patients retained their original ER status on recurrence. However although only half the ER +/+ tumours remained +/+, several tumours in other categories 'gained' receptor. Overall therefore the distribution of ER status in the recurrences is almost identical to that in the primary tumours.

*2c. Endocrine therapy*

The results from this subgroup of 32 patients (Table IIc) show a convincing trend for ER+/+ tumours to become negative following treatment with tamoxifen. Only one of 13 +/+ tumours remained +/+, this recurrence developing 20 months after the cessation of adjuvant tamoxifen therapy. This group accounts almost entirely for the difference in receptor variation between the results in Table I and Table IIa.

**Table II** The effect of intervening therapy on the ER status of patients with biopsies of their primary tumour (first assay) and first loco-regional recurrence (second assay)

| *First assay* | | *Second assay* *No. of patients in each category* | | | |
|---|---|---|---|---|---|
| *ER status* | *No.* | +/+ | 0/0 | +/0 | 0/+ |
| (a) With no intervening therapy | | | | | |
| +/+ | 41 | 33 | 8 | 0 | 0 |
| 0/0 | 40 | 6 | 33 | 1 | 0 |
| +/0 | 10 | 2 | 5 | 0 | 3 |
| 0/+ | 3 | 1 | 1 | 1 | 0 |
| Total | 94 | 42 | 47 | 2 | 3 |
| (b) Adjuvant chemotherapy | | | | | |
| +/+ | 10 | 5 | 2 | 2 | 1 |
| 0/0 | 13 | 1 | 10 | 0 | 2 |
| +/0 | 4 | 1 | 1 | 0 | 2 |
| 0/+ | 2 | 2 | 0 | 0 | 0 |
| Total | 29 | 9 | 13 | 2 | 5 |
| (c) Endocrine therapy | | | | | |
| +/+ | 13 | 1 | 12 | 0 | 0 |
| 0/0 | 14 | 1 | 13 | 0 | 0 |
| +/0 | 3 | 0 | 3 | 0 | 0 |
| 0/+ | 2 | 1 | 0 | 1 | 0 |
| Total | 32 | 3 | 28 | 1 | 0 |

*3. Patients with biopsies from their primary tumour and a recurrence in the contralateral breast*

As shown in Table III, the extent of receptor variation between the first and second tumours is no greater than is seen in the main group of loco-regional recurrences. Six of these women had received intervening adjuvant therapy (5 tamoxifen and 1 CMF) but this did not influence the results as 4 were initially 0/0 (3 remained 0/0 and 1 changed to +/+) and 2 were initially 0/+.

**Table III** ER status of sequential biopsies from primary breast tumours and recurrences developing in the contralateral breast

| *First assay* | | *Second assay* *No. of patients in each category* | | | |
|---|---|---|---|---|---|
| *ER status* | *No.* | +/+ | 0/0 | +/0 | 0/+ |
| +/+ | 10 | 8 | 1 | 0 | 1 |
| 0/0 | 14 | 4 | 10 | 0 | 0 |
| +/0 | 0 | 0 | 0 | 0 | 0 |
| 0/+ | 2 | 1 | 1 | 0 | 0 |
| Total | 26 | 13 | 12 | 0 | 1 |

*4. Changes in ER status in sequential biopsies of recurrent disease*

The pooled results from these 57 patients are shown in Table IV. Only 34 (59.9%) of them retained their original ER status on recurrence. Of the 19 +/+ tumours, 8 apparently lost receptor, becoming 0/0. Six of these 8 were receiving tamoxifen at the time of their second biopsy, one had just stopped CMF and only one had no intervening systemic therapy. None of the 11 who remained +/+ had received systemic treatment (7 had their first recurrence treated by excision alone and 4 by excision plus radiotherapy).

Thirty-one of these tumours were 0/0 on first recurrence and the majority, 23 (74.2%) remained 0/0. Three of the 7 who changed to +/+ were known to have been taking tamoxifen at the time of their first recurrence and had stopped this treatment by the time the second recurrence was biopsied. As with the primary tumours, none of the tumours whose first recurrence contained abnormal or incomplete receptor (+/0 and 0/+) retained this status on second recurrence (even those who had received no intervening therapy).

**Table IV** ER status of sequential biopsies from patients with recurrent disease (i.e. all patients with assays on a recurrence followed by a further recurrence)

| *First assay of recurrence* | | *Second assay of recurrence* *No. of patients in each category* | | | |
|---|---|---|---|---|---|
| *ER status* | *No.* | +/+ | 0/0 | +/0 | 0/+ |
| +/+ | 19 | 11 | 8 | 0 | 0 |
| 0/0 | 31 | 7 | 23 | 1 | 0 |
| +/0 | 4 | 0 | 4 | 0 | 0 |
| 0/+ | 3 | 3 | 0 | 0 | 0 |
| Total | 57 | 21 | 35 | 1 | 0 |

### Discussion

Several previous studies have shown conflicting data on the stability of cytosol ER status (Rosen *et al.*, 1977; Allegra *et al.*, 1980; Peetz *et al.*, 1982; Holdaway & Bowditch, 1983) but all of these studies were performed on relatively small numbers of patients. A number of more recent studies have also examined progesterone receptors in addition to cytosol ER in multiple tumour samples from individual patients (Harland *et al.*, 1983; Raemakers *et al.*, 1984; Nomura *et al.*, 1985) but none of these considered nuclear ER data. We have re-examined this question in terms of both cytosol and nuclear oestrogen receptors. While our data confirm that changes in ER status occur, the number of tumours changing spontaneously is small and in the absence of intervening therapy, cytosol and nuclear ER status is relatively stable.

The majority of tumours converting, particularly from ER-positive to ER-negative, have done so under the influence of systemic therapy. Tamoxifen appears to be the most consistent in this respect and, in this series, no tumour was found to have measurable oestrogen receptors if the patient was receiving tamoxifen at the time of biopsy. The precise mechanism of this action is unclear (Taylor *et al.*, 1982; Toma *et al.*, 1985). It may be simply that tamoxifen blocks the biochemical assay by competing with the radio-labelled oestradiol preventing it from binding to the receptor. Alternatively, in the longer term, it may be that tamoxifen interferes with receptor synthesis. Studies using monoclonal antibodies to oestrogen receptor for radioimmunoassay or histochemical staining may shortly resolve this point, though at present the expense of these antibodies is such that they are unlikely to be introduced widely for routine assays.

Both histochemical (King *et al.*, 1985) and biochemical (Silfversward *et al.*, 1980; Poulsen *et al.*, 1981) studies have shown considerable heterogeneity of oestrogen receptor distribution within breast tumours. An ER-positive tumour may therefore contain a significant population of ER-negative cells. It has therefore also been suggested that tamoxifen may selectively destroy ER-positive cells, leaving ER-negative cells to predominate in any subsequent recurrence. From our observations on sequential biopsies of recurrent disease however, we know that at least some tumours regain ER function after stopping treatment.

The effect of adjuvant chemotherapy on ER status is less well defined. Although a relatively high proportion of these tumours changed their status on recurrence, as many tumours appear to have gained ER as have lost ER, leaving the overall distribution of ER status in this group virtually unaltered on recurrence. There is no general agreement as to whether a recurrence in the opposite breast represents a true recurrence or a second primary tumour. The fact that 4 0/0 tumours out of 14 (see Table III) changed to +/+ on recurrence suggests that at least some of these tumours were new primaries rather than recurrences of the original tumour.

A further factor potentially influencing changes in ER status is the quality of the assay itself. All assays used in this study were subject to internal quality control and our laboratory actively participates in external quality control, through the EORTC receptor group (Koenders & Thorpe, 1983). The assay used in this laboratory and criteria for determining receptor concentration were established over ten years ago (Laing *et al.*, 1976). All the samples in this study fulfil these criteria and we therefore feel that experimental error has not significantly contributed to the changes in ER status observed.

Despite having histological evidence of breast cancer in all the tumours assayed, a number of samples of 'unexpected' negative ER results occurred in samples with very low DNA levels. This problem is particularly relevant with cutaneous local recurrences, where the proportion of the sample composed of tumour cells may be very small. Although we do not set a lower limit for acceptable DNA level we routinely include the tumour DNA concentration in our reports to clinicians and comment if this is low. The introduction of immunohistochemical methods of localising oestrogen receptor should help to identify some of these ER-positive tumours of low cellularity (King *et al.*, 1985).

We conclude that in the absence of intervening systemic therapy, ER status of breast tumours is relatively stable and the ER status of an adequate sample from the primary tumour should predict the likelihood of response to endocrine therapy when the tumour recurs.

### References

ALLEGRA, J.C., BARLOCK, A., HUFF, K.K. & LIPPMAN, M.E. (1980). Changes in multiple or sequential estrogen receptor determinations in breast cancer. *Cancer*, **45**, 792.

CAMPBELL, F.C., BLAMEY, R.W., ELSTON, C.W., MORRIS, A.N., NICHOLSON, R.I., GRIFFITHS, K. & HAYBITTLE, J.L. (1981). Quantitative oestradiol receptor values in primary breast cancer and response of metastases to endocrine therapy. *Lancet*, **ii**, 1317.

COWAN, S., LOVE, C. & LEAKE, R.E. (1984). The value of determination of nuclear oestrogen receptors in breast cancer biopsies. *Recent Res. Cancer Res.*, **91**, 50.

CRAWFORD, D.J., COWAN, S.K., McMENAMIN, M., HYDER, S., SMITH, D.C. & LEAKE, R.E. (1984). A new storage procedure for human tumour biopsies prior to oestrogen receptor measurement. *Cancer Res.*, **44**, 2348.

HARLAND, R.N.L., BARNES, D.M., HOWELL, A., RIBEIRO, G.G., TAYLOR, J. & SELLWOOD, R.A. (1983). Variation of receptor status in cancer of the breast. *Br. J. Cancer*, **47**, 511.

HOLDAWAY, I.M. & BOWDITCH, J.V. (1983). Variation in receptor status between primary and metastatic breast cancer. *Cancer*, **52**, 479.

KATZENELLENBOGEN, B.S. & LEAKE, R.E. (1974). Distribution of the oestrogen-induced protein and of total protein between endometrial and myometrial fractions of the immature rat uterus. *J. Endocrinol.*, **63**, 439.

KING, W.J., DE SOMBRE, E.R., JENSEN, E.V. & GREENE, G.L. (1985). Comparison of immunocytochemical and steroid binding assays for estrogen receptor in human breast cancers. *Cancer Res.*, **45**, 293.

KOENDERS, T. & THORPE, S.M. (1983). Standardization of steroid receptor assays in human breast cancer. 1: Reproducibility of oestradiol and progesterone receptor assays. *Eur. J. Cancer Clin. Oncol.*, **19**, 1221.

LAING, L., CALMAN, K.C., SMITH, D.C. & LEAKE, R.E. (1976). Oestrogen binding protein in plasma of breast cancer patients. *Lancet*, **ii**, 745.

LEAKE, R.E., LAING, L. & SMITH, D.C. (1979). A role for nuclear oestrogen receptors in prediction of therapy regime for breast cancer patients. In *Steroid Receptor Assays in Human Breast Tumours: Methodological and Clinical Aspects*, King, R.J.B. (ed) p. 73. Alpha Omega Press: Cardiff.

LEAKE, R.E., LAING, L., CALMAN, K.C., MACBETH, F.R., CRAWFORD, D.J. & SMITH, D.C. (1981). Oestrogen receptor status and endocrine therapy of breast cancer: Response rates and status stability. *Br. J. Cancer*, **43**, 59.

LOVE, C.A., COWAN, S.K., LAING, L.M. & LEAKE, R.E. (1983). Stability of human nuclear oestrogen receptor: influence of temperature and ionic strength. *J. Endocrinol.*, **99**, 423.

LOWRY, O., ROSENBROUGH, N., FARR, A. & RANDALL, R. (1951). Protein measurement with the Folin phenol reagent. *J. Biol. Chem.*, **193**, 265.

McGUIRE, W.L., CARBONE, P.P., SEARS, M.E. & ESCHER, G.C. (1975). Estrogen receptors in human breast cancer: an overview. In *Estrogen Receptors in Human Breast Cancer*, Mcguire, W.L. *et al.*, (eds) p. 1. Raven Press: New York.

NOMURA, Y., TASHIRO, H. & SHINOZUKA, K. (1985). Changes of steroid hormone receptor content by chemotherapy and/or endocrine therapy in advanced breast cancer. *Cancer*, **55**, 546.

PEETZ, M.E., NUNLEY, D.L., MOSELEY, H.S., KEENAN, E.J., DAVENPORT, C.E. & FLETCHER, W.S. (1982). Multiple simultaneous and sequential estrogen receptor values in patients with breast cancer. *Am. J. Surg.*, **143**, 591.

POULSEN, H.S., JENSEN, J. & HERMONSEN, C. (1981). Human breast cancer: heterogeneity of oestrogen binding sites. *Cancer*, **48**, 1791.

RAEMAKERS, J.M., BEEX, L.V., KOENDERS, A.J., PIETERS, G.F., SMALS, A.G., BENRAAD, T.J. & KLOPPENBORG, P.W. (1984). Concordance and discordance of estrogen and progesterone receptor content in sequential biopsies of patients with advanced breast cancer: Relation to survival. *Eur. J. Cancer Clin. Oncol.*, **20**: 1011.

ROSEN, P.P., MENENDEZ-BOTET, C.J., URBAN, J.A., FRACCHIA, A. & SCHWARTZ, M.K. (1977). Estrogen receptor protein (ERP) in multiple tumour specimens from individual patients with breast cancer. *Cancer*, **39**, 2194.

SILFVERSWARD, C., SKOOG, L., HUMLA, S., GUSTAFFSEN, S.A. & NORDENSKJOLD, B. (1980). Intratumoral variation in cytoplasmic and nuclear oestrogen receptor concentrations in human mammary carcinoma. *Eur. J. Cancer*, **16**, 59.

TAYLOR, R.E., POWLES, T.J., HUMPHREYS, J. & 5 others (1982). Efects of endocrine therapy on steroid receptor content of breast cancer. *Br. J. Cancer*, **45**, 80.

TOMA, S., LEONESSA, F. & PARIDAENS, R. (1985). The effects of therapy on estrogen receptors in breast cancer. *J. Steroid Biochem.*, **23**: 1105.